# Hypertrophic Lichen Planus: A Precursor Lesion for Squamous Cell Carcinoma

**DOI:** 10.7759/cureus.55450

**Published:** 2024-03-03

**Authors:** Sowmiya M, Thanka Johnson, Manimaran R, Srismitha S, Janhavi M S

**Affiliations:** 1 Pathology, Sree Balaji Medical College and Hospital, Chennai, IND; 2 Plastic Surgery, Sree Balaji Medical College and Hospital, Chennai, IND

**Keywords:** scc screening, malignant transformation, scc precursor, non-healing ulcer, hypertrophic lichen planus, cutaneous squamous cell carcinoma

## Abstract

Squamous cell carcinoma (SCC) is the second most common malignant tumor of the skin. This case report aims to report a case of cutaneous squamous cell carcinoma in an elderly male presenting with a non-healing ulceroproliferative growth on the shin of the right lower limb and a hypopigmented patch on the shin of the left lower limb. The significant feature of this case is that in the shin of the left lower limb, SCC appears in the background of chronic hypertrophic lichen planus (HLP) but in the right lower limb, there is no evidence of a background hypertrophic lichen planus. There are only a few similar cases reported so far in the literature showing long-standing hypertrophic lichen planus as a risk factor for the development of cutaneous squamous cell carcinoma. This case illustrates that chronic hypertrophic lichen planus should be considered as a potential precursor lesion for SCC. Regular screening is essential for early detection, enabling timely intervention for improving patient outcomes.

## Introduction

Most squamous cell carcinomas (SCCs) occur in sun-exposed areas of the skin. It is defined as the carcinoma of keratinocytes that infiltrates the dermis. The Global Burden of Disease Study (GBD) provides a systematic standardized approach for the assessment of major diseases, risk factors, and clinical outcomes. The following epidemiological data are collected from the latest 2019 GBD update. The estimated global incidence of SCC and global deaths due to SCC were 2,402,221 and 56,054, respectively. Its incidence rate is estimated to be highest in Canada (1600 cases per 100,000). In India, the burden of SCC is relatively low, with prevalence and incidence rates of 0.13 and 0.27 per 100,000, respectively [1]. The most common risk factor is ultraviolet radiation and other risk factors are older age, immunosuppressive treatment, ionizing radiation, other carcinogens, and smoking. They can also arise from sites of chronic inflammation from burn scars, chronic ulcers, sinus tracts, or inflammatory dermatoses. Other rare cases are due to inherited disorders such as xeroderma pigmentosum, dystrophic epidermolysis bullosa, epidermodysplasia verruciformis, and human papillomavirus infection [2].

Ultraviolet radiation by the induction of DNA damage causing mutations in epidermal keratinocytes contributes to the development of actinic keratosis (AK) and SCC. Mutations in the p53 tumor suppressor gene have been detected in 30 -50% of skin specimens from patients with AK and SCC [3]. Epidemiological studies have estimated that about 60% of SCCs arise from pre-existing AK [4,5]. Though the majority of SCCs are considered relatively less aggressive, about 5% can metastasize. These advanced SCCs with metastatic involvement have a poor prognosis, with an estimated five-year survival rate of less than 30%. Newer diagnostic modalities like gene expression profiling have been validated to predict tumor aggressiveness and metastatic probability. Programmed cell death protein 1 (PD1) inhibitor cemiplimab has been shown to improve the remission rates of advanced tumors [6].

Hypertrophic lichen planus (HLP) is a chronic variant of lichen planus (LP) favoring the lower extremities and showing prominent epidermal hyperplasia and hyperorthokeratosis. It is characterized by lichenoid mononuclear inflammatory cells infiltrating the upper dermis. It presents as pruritic hyperkeratotic or verrucous plaques [7]. The majority of squamous cell carcinoma is found to occur in HLP lesions located on the lower limbs. A retrospective chart review and database analysis showed only 38 cases of squamous cell carcinoma arising in hypertrophic lichen planus in 16 women and 22 men [8].

## Case presentation

**Figure 1:**
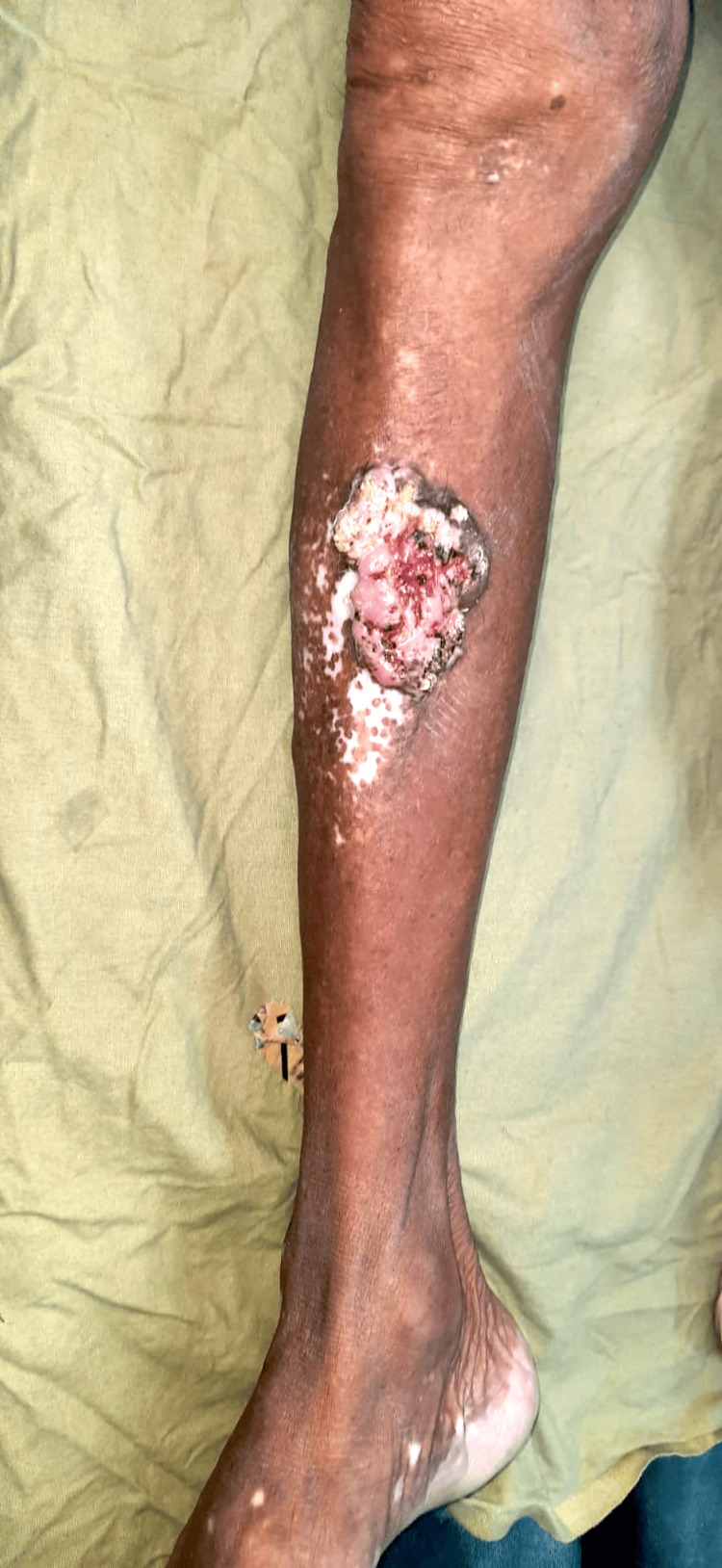
Ulceroproliferative lesion over the anterior aspect of the right lower limb

**Figure 2:**
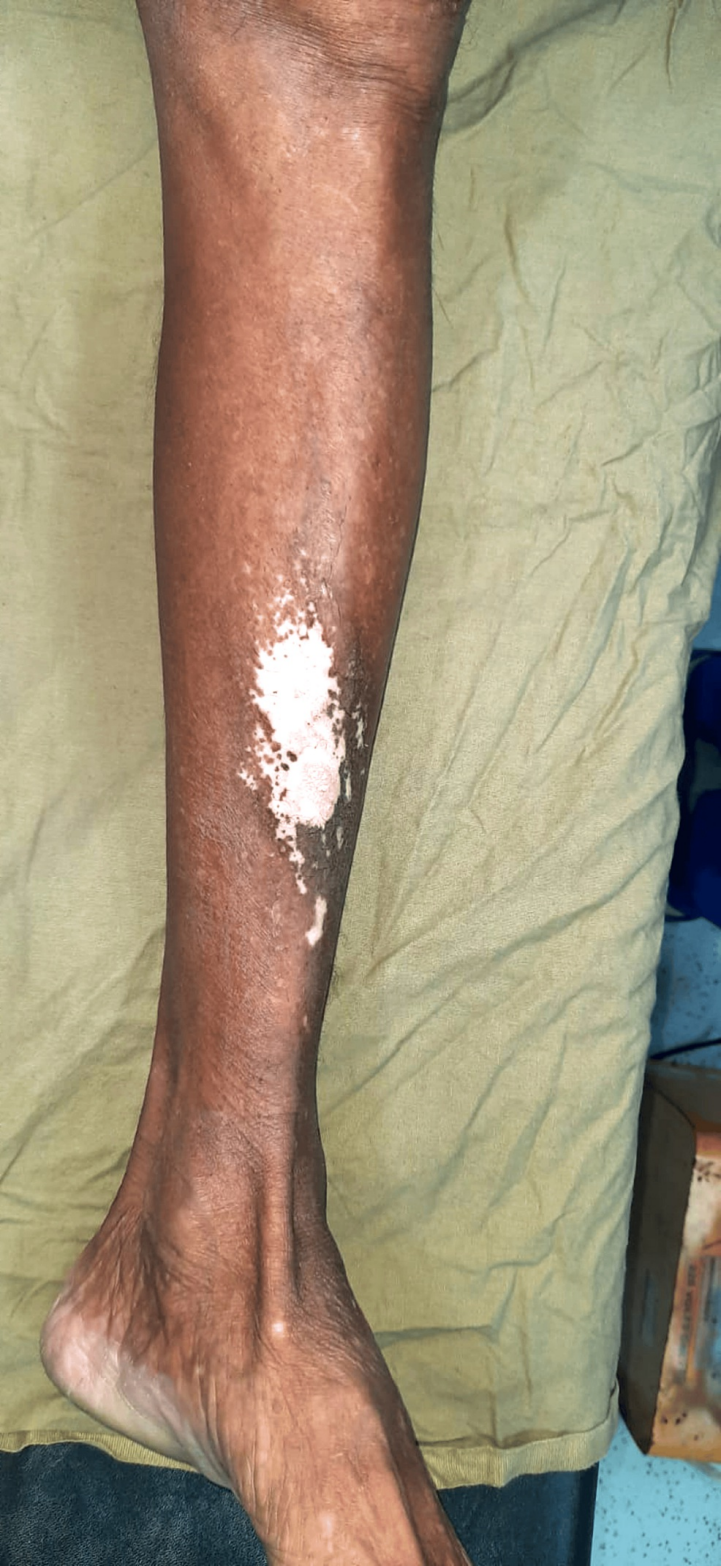
Hypopigmented patch over the anterior aspect of the left lower limb

An 83-year-old male presented with complaints of swelling and pain over the right leg for four years and skin discoloration over the left leg for four years. The patient had a history of lichen planus on the shin of the left leg and was on topical treatment. There was no history of diabetes, burns, or any autoimmune disorder. On local examination, there was an ulceroproliferative lesion on the anterior aspect of the shin of the right leg about 7 x 5 cm in size, about 7 cm below the knee. The margins of the lesion were irregular with raised everted edges, with areas of active bleeding from the ulceration. The lesion was surrounded by scattered areas of hypopigmented patches (Figure 1). On the anterior aspect of the shin of the left leg, there was a hypopigmented patch about 7 x 5 cm (Figure 2). There were no palpable regional lymph nodes.

Under sterile aseptic precaution, both lesions were removed by wide local excision and sent for histopathological examination. On gross examination, the excised specimen from the right leg was measured to be 9 x 6 x 2 cm. The external surface showed an ulceroproliferative lesion measuring 7 x 5 cm. The cut surface was gray-white in color and friable in consistency. The lesion was 1.5 cm from the superior margin, 1.2 cm from the lateral margin, 1.5 cm from the inferior margin, 1 cm from the medial margin, and 0.1 cm from the deep resected margin. The excised specimen from the left leg was measured to be 12 x 4 x 0.5 cm. The external surface showed a gray-white lesion measuring 5.5 x 2 cm. The cut surface was gray-white in color and firm in consistency. The lesion was 0.5 cm from the medial margin, 1 cm from the lateral margin, 0.5 cm from the superior margin, 0.3 cm from the inferior margin, and 0.2 cm from the deep resected margin.

**Figure 3:**
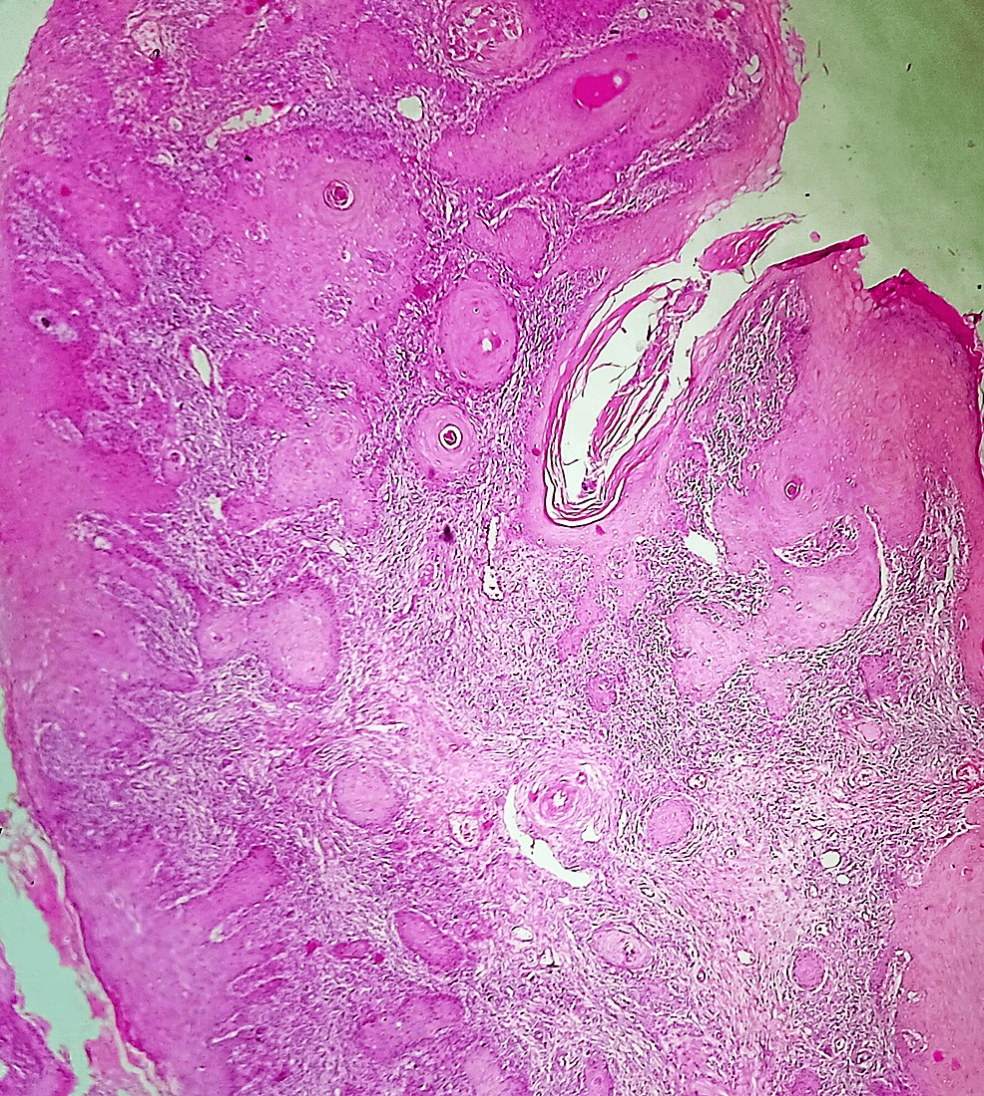
Invasive squamous cell carcinoma The tumor consists of irregular masses of squamous cells that have invaded the dermis (H&E, x100). H&E, hematoxylin and eosin

A microscopic histopathological examination of formalin-fixed, paraffin-embedded tissue sections stained with hematoxylin and eosin (H&E) was done. Sections from the lesion on the right leg showed features of well-differentiated squamous cell carcinoma (Figure 3) and the surrounding tumor-free margins were measured to be 1.5 cm superiorly, 1.2 cm laterally, 1.5 cm inferiorly, and 1 cm medially. The deep resected margin was 1 mm from the tumor and there is no evidence of lymphovascular invasion and perineural invasion.

**Figure 4:**
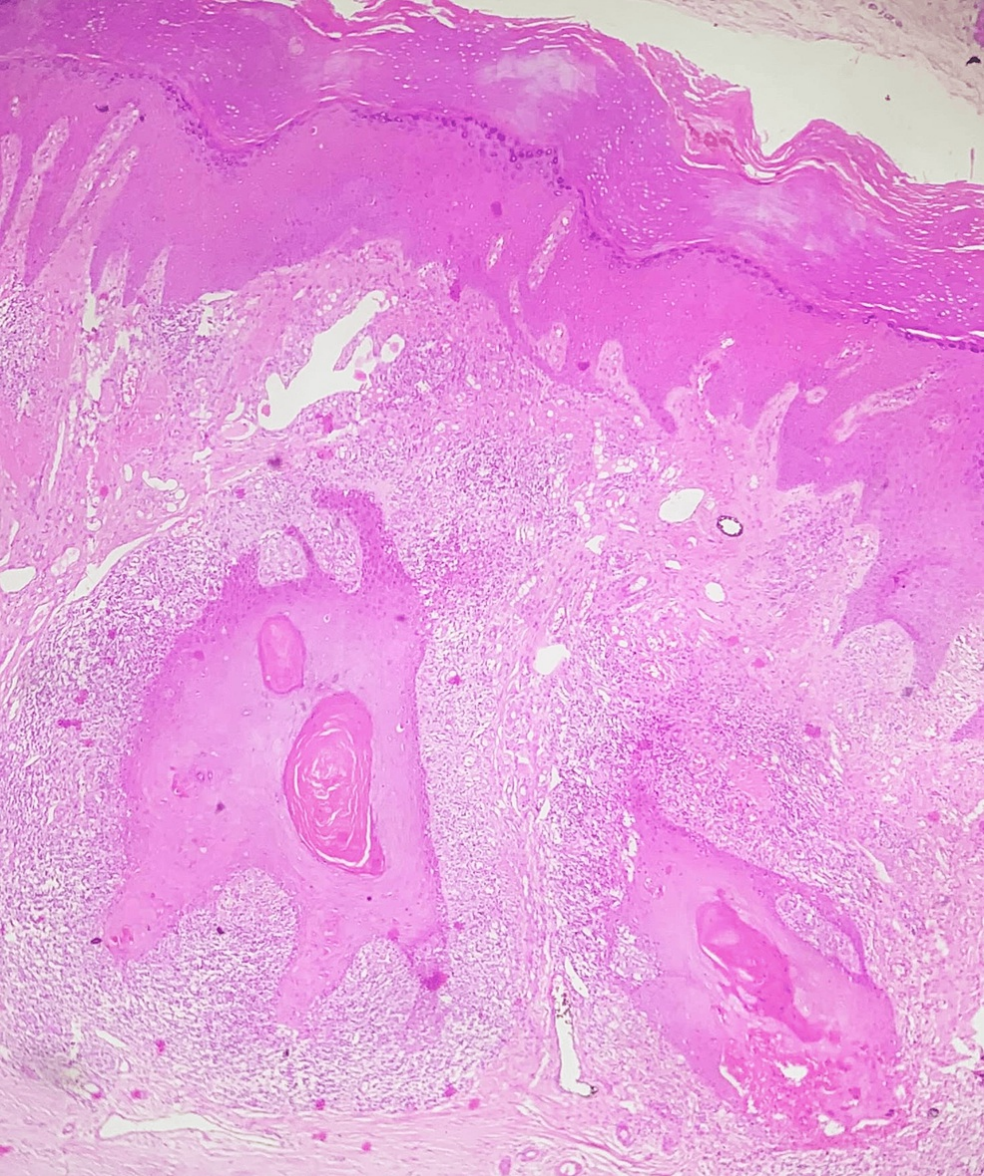
Squamous cell carcinoma arising in the background of hypertrophic lichen planus (H&E, x100) H&E, hematoxylin and eosin

Sections from the lesion on the left leg showed hyperkeratosis, irregular acanthosis, and lichenoid mononuclear inflammatory cell infiltration of the upper dermis. There was basal cell degeneration and hypergranulosis. Occasional Civatte bodies were also present. However, there was a focus on well-differentiated squamous cell carcinoma (Figure 4) measuring 4 mm in size as measured microscopically.

The histopathological diagnosis of well-differentiated squamous cell carcinoma was made for the lesion on the right leg. The lesion on the left leg showed histological features consistent with that of hypertrophic lichen planus along with well-differentiated squamous cell carcinoma measuring 4 mm.

## Discussion

In this case, the patient is an elderly male who is a farmer. Both age and occupation, predisposing to extended periods of sun exposure, are known risk factors for cutaneous SCC. In addition, SCC is also known to occur at sites of scaring and chronic inflammation. In this patient, one of the SCCs occurred in a background of chronic HLP. The literature on SCC arising from HLP is scarce, with fewer than 50 reported cases [8]. The exact pathogenesis linking HLP and SCC remains unclear. One proposed mechanism is that a persistent inflammatory milieu associated with HLP has the potential to induce genetic changes and disrupt cell cycle regulation, ultimately predisposing to the development of malignancy [9]. The development of an aggressive form of highly invasive SCC with widespread metastasis from long-standing HLP has been reported [10]. This case illustrates the significance of contemplating chronic HLP as a potential precursor lesion for SCC, as it emphasizes the importance of regular screening in early detection and timely intervention enabling favorable outcomes.

The average time between the diagnosis of HLP and the development of SCC is about 10 years as per the cases reported [11]. In this case, the diagnosis of HLP was made four years back, on which SCC has developed. Clinicians should be aware of this rare association and anticipate SCC in any chronic inflammatory dermatoses and perform screening to rule out SCC. Suspicious changes, such as ulceration, rapid growth, or bleeding, are red flags and warrant further investigation. Newer diagnostic modalities using gene expression profiling with a panel of 40 genes have been validated to predict tumor aggressiveness and metastatic risk, thus guiding management strategies [12]. In this case, early detection and timely intervention by wide local excision of the lesions followed by a skin-to-skin graft helped in healthy healing. The patient is on regular follow-up with no evidence of recurrence.

Though most SCCs are relatively less aggressive, 5% of SCCs become metastatic, which carries a very poor prognosis. Recently, neoadjuvant cemiplimab is currently in phase 2 trial and has been found to be effective for advanced SCC [13]. Thus early detection and management are crucial to improve patient outcomes and prevent complications. Further research is warranted to unravel the intricate interplay between chronic inflammation and carcinogenesis.

## Conclusions

The association between hypertrophic lichen planus and the development of squamous cell carcinoma is not well understood. The significance of HLP as a potential precursor lesion for SCC is undermined due to the paucity of literature. This often leads to late diagnosis and metastatic complications. Therefore, the malignant transformation of chronic HLP should be anticipated. The risk of predisposition to malignancy should be educated to the patient and the importance of regular follow-up should be emphasized in order to avoid the complications of late diagnosis. Thus, screening for high-risk features such as proliferative growth, non-healing ulceration and spontaneous bleeding from the site of long-standing HLP is vital.
